# Adhesion and Removal of Thirdhand Smoke from Indoor Fabrics: A Method for Rapid Assessment and Identification of Chemical Repositories

**DOI:** 10.3390/ijerph18073592

**Published:** 2021-03-30

**Authors:** Giovanna L. Pozuelos, Peyton Jacob, Suzaynn F. Schick, Esther E. Omaiye, Prue Talbot

**Affiliations:** 1 Department of Molecular, Cell and Systems Biology, University of California, Riverside, CA 92521, USA; gpozu001@ucr.edu (G.L.P.); esther.omaiye@ucr.edu (E.E.O.); 2 Department of Medicine, Division of Cardiology, Clinical Pharmacology Program, University of California San Francisco, San Francisco, CA 94110, USA; peyton.jacob@ucsf.edu; 3 Department of Medicine, Division of Occupational and Environmental Medicine, University of California San Francisco, San Francisco, CA 94110, USA; suzaynn.schick@ucsf.edu

**Keywords:** thirdhand smoke, nicotine, tobacco, assays, remediation

## Abstract

Thirdhand smoke (THS) is an environmental contaminant that may cause adverse health effects in smokers and nonsmokers. Currently, time-consuming analytical methods are necessary to assess chemicals in THS repositories, like upholstered furniture and clothing. Our goal was to develop a rapid, accessible method that can be used to measure THS contamination in common household fabrics and to evaluate remediation. Cotton, terry cloth, polyester, and wool were exposed to THS for various times in a controlled laboratory environment and then extracted in various media at room temperature or 60 °C to develop an autofluorescent method to quantify THS. Concentrations of nicotine and related alkaloids in the extracts were determined using liquid chromatography–tandem mass spectrometry (LC-MS/MS) and high-performance liquid chromatography (HPLC). The autofluorescence of extracts was proportional to the time and amount of THS exposure received by cotton and terry cloth. Extracts of polyester and wool did not show autofluorescence unless heat was applied during extraction. Nicotine, nicotine alkaloids, and TSNA concentrations were higher in THS extracts from cotton and terry cloth than extracts of polyester and wool carpet, in agreement with the autofluorescence data. For fabrics spiked with 10 mg of nicotine, extraction efficiency was much higher from terry cloth (7 mg) than polyester (0.11 mg). In high relative humidity, nicotine recovery from both cotton and polyester was 80% (~8 mg). Our results provide a simple, rapid method to assess THS contaminants in household fabrics and further show that THS extraction is influenced by fabric type, heat, and humidity. Thus, remediation of THS environments may need to vary depending on the fabric reservoirs being treated. Understanding the dynamics of THS in fabrics can help set up appropriate remediation policies to protect humans from exposure.

## 1. Introduction

Thirdhand smoke (THS) is an environmental pollutant that can be absorbed through dermal contact, inhalation, or ingestion by both smokers and nonsmokers. THS comprises secondhand smoke from the burning end of a cigarette plus exhaled mainstream smoke that settles on indoor surfaces where the residue can remain, react, be re-emitted, or be resuspended for months or years after smoking has ceased [1,2]. THS is a mixture of hazardous volatile and semivolatile organic chemicals, polycyclic aromatic hydrocarbons, metals, and secondary compounds generated through reactions with atmospheric pollutants (e.g., ozone and nitrous acid) [3]. Nicotine, a major component of THS, can react with ambient concentrations of oxidants like nitrous acid or ozone to form carcinogenic tobacco-specific nitrosamines (TSNAs), such as 4-(methylnitrosamino)-1-(3-pyridinyl)-1-butanone (NNK), and N-nitrosonornicotine (NNN) [2,3,4,5]. Concentrations of TSNAs can increase as THS ages, increasing the toxicity of THS over time [5,6].

THS causes genotoxic damage and a stress-induced cellular defense response in cultured cells [7,8]. Alterations in DNA were found to occur in cells from mice and humans during both short- and long-term exposure to THS [7]. Mouse neural stem cells undergo morphological changes such as blebbing, fragmentation, cytoskeletal disruption, and vacuolization when treated with aqueous THS extracts [9,10]. THS also causes stress-induced mitochondrial hyperfusion (SIMH) and increases in mitochondrial membrane potential, ATP levels, and reactive oxygen species (ROS) [8]. In a metabolomics study, male germ cells exposed to THS downregulated molecular pathways involved in nucleic acid and ammonia metabolism and upregulated glutathione metabolism [11]. Three-week-old mice exposed to THS fabric for 6 months showed an increase in inflammatory cytokines in lung tissue and impaired wound healing [12]. Mice also became insulin resistant as a result of oxidative stress [13]. Oxidative stress in skeletal muscle cells and accumulation of H_2_O_2_ accompanied with low catalase activity were also found in exposed mice [13]. Hang et al. (2017) showed that THS-exposed neonatal and adult mice had changes in hematopoiesis [14].

Unlike secondhand smoke exposure, which occurs mainly through inhalation, human exposure to THS can also include ingestion and absorption through the skin [15,16]. Children and toddlers are particularly vulnerable to THS due to their low body mass and frequent contact with indoor surfaces during crawling and mouthing. A toddler mouthing a small piece of fabric exposed to THS can be exposed to 2.2 μg of nitrosamines/day, a concentration approximately 16-fold greater than the inhalation exposure of an adult passive smoker (0.14 μg/day) [6]. In addition, young children have a high respiratory rate relative to their body weight, which increases the amount of THS they can inhale when compared to adults [17]. Recently, we showed that acute inhalation of THS alters the nasal epithelium transcriptome of nonsmoking women in a manner consistent with stress-induced mitochondrial hyperfusion (SIMH), demonstrating that even short exposure to THS produces adverse effects in humans [18].

THS toxicants are found in both smoking and nonsmoking environments [19,20]. Infants whose mothers smoked outdoors to protect their children from exposure still had much higher levels of cotinine, a nicotine metabolite, in their urine than infants of nonsmoking parents [21]. Detectable concentrations of THS were found on incubators and cribs from neonatal intensive care units (ICU) in hospitals. ICU infants showed detectable levels of cotinine and NNAL, a metabolite of NNK [16]. THS can be absorbed in the clothes of smokers and nonsmokers who are exposed to SHS [19]. Sheu et al. 2020 showed that THS contaminants can be transported into a smoking-free movie theater by moviegoers’ clothing in quantities equivalent to those found in secondhand smoke generated from 1–10 cigarettes [22]. The widespread distribution of THS in indoor environments, such as movie theaters, hospitals, and hotels, makes nonsmokers susceptible to THS exposure.

THS and its potential toxicity demonstrate a need to (1) develop rapid, accessible methods to quantify THS so that it can be compared across laboratories and field sites, (2) measure THS retention in indoor fabric reservoirs, and (3) determine how to best remediate indoor environments, especially when there is a need to protect nonsmokers and vulnerable populations. One goal of this paper is to introduce a rapid method based on autofluorescence of chemicals in smoke that permits cross-comparisons of THS extracts from different samples, laboratories, and environments. With this method, THS laboratories will be able to normalize different THS extracts so that comparisons between batches can be made. There is currently not a rapid method for making such comparisons. THS extracts are generally evaluated using either LC-MS/MS or HPLC, which require a trained specialist to prepare samples and analyze data. The fluorescence spectrophotometer is easy to operate, does not require special training, can be used to perform analysis in minutes, and is much less costly than the LC-MS/MS and HPLC. The autofluorescence method is also easily adaptable to most labs with minimal expense. Additional goals were to compare the release of THS chemicals from various household fabric repositories and determine the factors that affect the extraction of THS and remediation of indoor environments. We tested the sorption of THS chemicals using household fabrics that are not frequently washed and would therefore likely be long-term repositories of THS. These included upholstery cotton, terry cloth, upholstery polyester, and wool carpet that were exposed to THS in a controlled laboratory environment for 1, 6, 12, or 18 months.

## 2. Materials and Methods

### 2.1. Exposure of Household Fabrics to Firsthand and Secondhand Cigarette Smoke

Cotton, terry cloth, polyester, and wool carpet were purchased in Riverside, California, and sent to Dr. Suzaynn Schick at the University of California, San Francisco, where they were impregnated with THS as previously described [23]. Briefly, Marlboro Red cigarettes (Philip Morris Co., Richmond, VA, USA) were smoked using an automatic smoking machine (Model TE-10z smoking machine, Teague Enterprises, Woodland, CA, USA). The smoke was diluted with conditioned and filtered air and passed through a 6-m^3^ stainless steel smoke aging chamber where the fabrics were hung [24]. Airflow through the chamber was measured using an air velocity transmitter (model 641-b Dwyer Instruments, Michigan City, IN). Particle concentration was measured using a laser photometer (Dusttrak II, model 8530, TSI Inc., Shoreview, MI) located in the ducting immediately before the aging chamber. The milligrams of particulate material and the hours of smoke exposure for each time interval (1, 6, 12, or 18 months) are shown in Table 1 and Supplementary Tables S1–S4, which contain additional information on days when exposures were done.

Pieces (11″ × 8″) of cotton, terry cloth, polyester, and wool carpet were exposed to different concentrations of particulate material derived from smoke for 1, 6, 12, or 18 months. Prior to exposure, fabrics were washed three times in a domestic washing machine using an unscented, enzyme-free laundry detergent (Country Save, Arlington, WA, USA) in hot water with two rinses/cycle after each wash. After a fourth and final wash with no detergent, fabrics were line-dried. After exposure, fabrics were stored in tightly sealed Mylar bags at −20 °C and shipped to UCR on dry ice. Once received, they were stored at −80 °C until used.

### 2.2. Preparation of THS Aqueous Extracts for Fluorescence Intensity and LC-MS/MS Analysis

Aqueous extractions of THS and unexposed control fabric (stored in a smoke-free laboratory) were prepared in serum-free keratinocyte medium (GIBCO 17005-042) supplemented with keratinocyte supplements (Gibco 37000-015), PBS (−), or dimethyl sulfoxide (DMSO). THS-exposed and unexposed fabrics were soaked at a ratio of 0.1 g of fabric/mL of cell medium, PBS (−), or DMSO on a rotating shaker for 1 h at room temperature. For heated extractions, 15 mL conical tubes containing fabric and 3 mL of buffer were placed on a heating block for 1 h at 60 °C. Extract was recovered from fabric using a 30 mL Luer Henke Syringe (Henke Sass Wolf, Germany) and sterilized by passing through a 0.22 μM filter (Pall Corporation, Port Washington, NY, USA). The extract was aliquoted and stored at −80 °C for further use.

### 2.3. Fluorescence Intensity

Samples (200 µL) of extracts of cotton, terry cloth, polyester, and wool carpet in DMSO, PBS, and cell medium were added to a 96-well plate. Fluorescence intensity was measured at excitation/emissions of 360/460, 485/528, and 540/590 using a Bio-Tek Synergy HTX multimode microplate reader. Control extracts of cloth samples without THS exposure were measured in triplicate.

### 2.4. LC-MS/MS Analysis of Nicotine, Tobacco Alkaloids, and TSNAs

Quantification of nicotine, other tobacco alkaloids (cotinine, myosmine, N-formylnornicotine, nicotelline, bipyridine), and TSNAs in PBS was done at the University of California UCFS by liquid chromatography coupled with tandem mass spectrometry (LC-MS/MS) as previously described [25]. The limits of quantitation for each of the chemicals analyzed are as follows: nicotine: 1.02 ng/mL; myosmine: 0.305 ng/mL; 2,39-bipyridine: 0.914 ng/mL; cotinine: 0.914 ng/mL; N-formylnornicotine: 0.305 ng/mL; nicotelline: 0.030 ng/mL; NNN: 0.030 ng/mL; NNK: 0.0130 ng/mL; NNA: 0.010 ng/mL.

### 2.5. Exposure to Pure Nicotine

Samples of cotton, terry cloth, and polyester were exposed to pure nicotine to test factors affecting autofluorescence. Fabrics were exposed at room temperature to 10 mg of nicotine (Sigma, St. Louis, MO, USA) in 50 µL droplets of purified water (10 mg/50 µL) for 24 h at room temperature. During the 24-h incubation, an additional piece of cotton fabric was placed underneath each treated fabric to test for fabric permeability. The cotton fabric served as a reservoir for residual nicotine that soaked through the exposed fabrics. A set of fabrics were also treated for a short incubation period of 3 h at room temperature. In addition, a separate 24-h exposure experiment was performed in a tightly sealed vial to minimize air drying and increase humidity.

### 2.6. Preparation of Aqueous Extracts from Nicotine-Treated Fabrics for HPLC Analysis

After exposure, fabrics were cut and soaked in 3 mL of HPLC unbuffered mobile phase made of 76.9% water, 23% acetonitrile, and 0.1% triethylamine. Extraction efficiency was determined by soaking the same fabric in buffer three consecutive times for a duration of 3 min each in 15 mL conical tubes. For heated extractions, 15 mL conical tubes containing fabric and 3 mL unbuffered mobile phase were placed on a heating block for 1 h at 60 °C. After incubation, extracts were sterilized with a 0.22 μM filter (Pall Corporation, Port Washington, NY, USA).

### 2.7. HPLC Analysis of Nicotine

The high-performance liquid chromatography (HPLC) method for quantifying nicotine in fabric was adopted from the calibration protocol described previously [26]. The accuracy and precision of the calibration curve were validated by injecting three samples of nicotine, 333, 500, and 637 µg/mL, and determining the percent error at each concentration. For each concentration, the error was <1% (0.681% for 333 µg/mL, 0.319% for 500 µg/mL, and 0.654% for 637 µg/mL). Periodic validation of the calibration curve was performed to ensure there were no changes or drift.

Nicotine concentrations in fabric extract were analyzed using a Hewlett Packard Series 1100 HPLC with adjustments to a method previously described [26]. Since the extraction was performed in the unbuffered mobile phase, no further dilution was required. For the analysis, 100% extracts of the exposed fabric were used. Care was taken to accurately aspirate the solution so as not to introduce air bubbles. The injection volume for all samples was 5 μL. The limit of quantification for nicotine was 10 μg/mL, with a limit of detection of 50 ng/mL. Each sample was injected and analyzed three times. The values reported are the means and standard deviations of three independent experiments.

## 3. Results

### 3.1. THS Fabric Exposure

**Figure 1:**
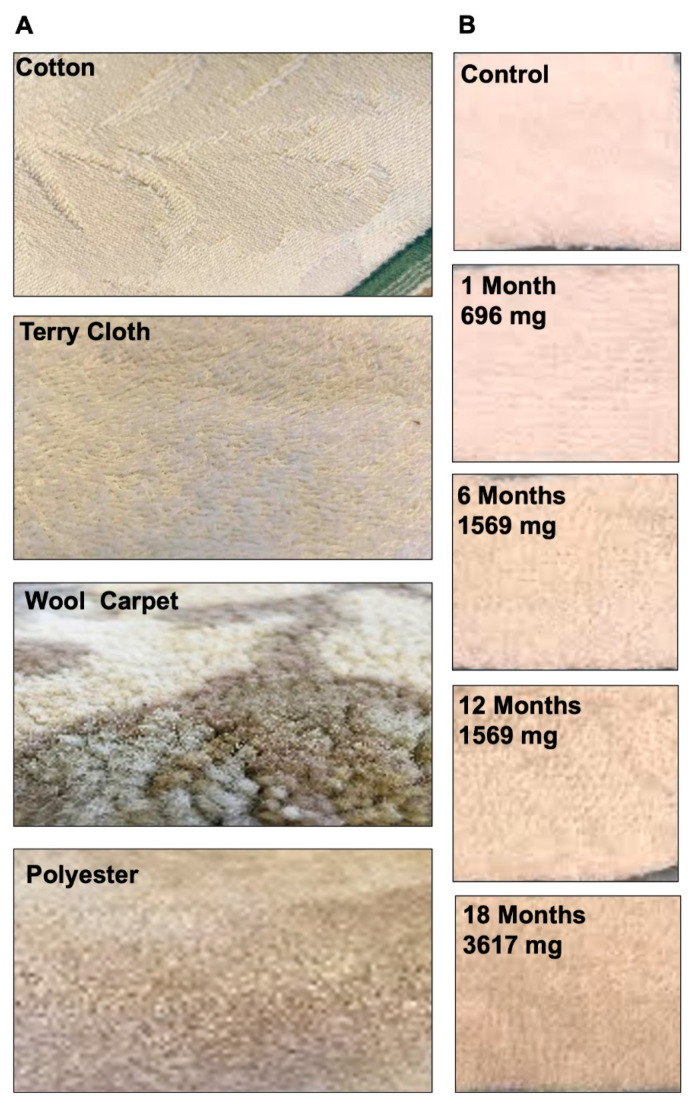
Common household fabrics exposed to thirdhand smoke (THS). (**A**) Cotton, terry cloth, wool carpet, and polyester were exposed to THS. All samples in A are unexposed fabrics. (**B**) Terry cloth exposed to different concentrations (mg) of particulate material from cigarette smoke at 1, 6, 12, and 18 months.

Cotton, terry cloth, polyester, and wool carpet were exposed for 1, 6, 12, and 18 months to 696, 1569, 1795, and 3617 mg of total particulate material, respectively (Figure 1A shows unexposed fabrics; Table 1). THS accumulation can be visualized in terry cloth, which gradually turned brown with increased exposure (Figure 1B).

### 3.2. Autofluorescence of THS Extracts Is Directly Related to the Length of Exposure

**Figure 2:**
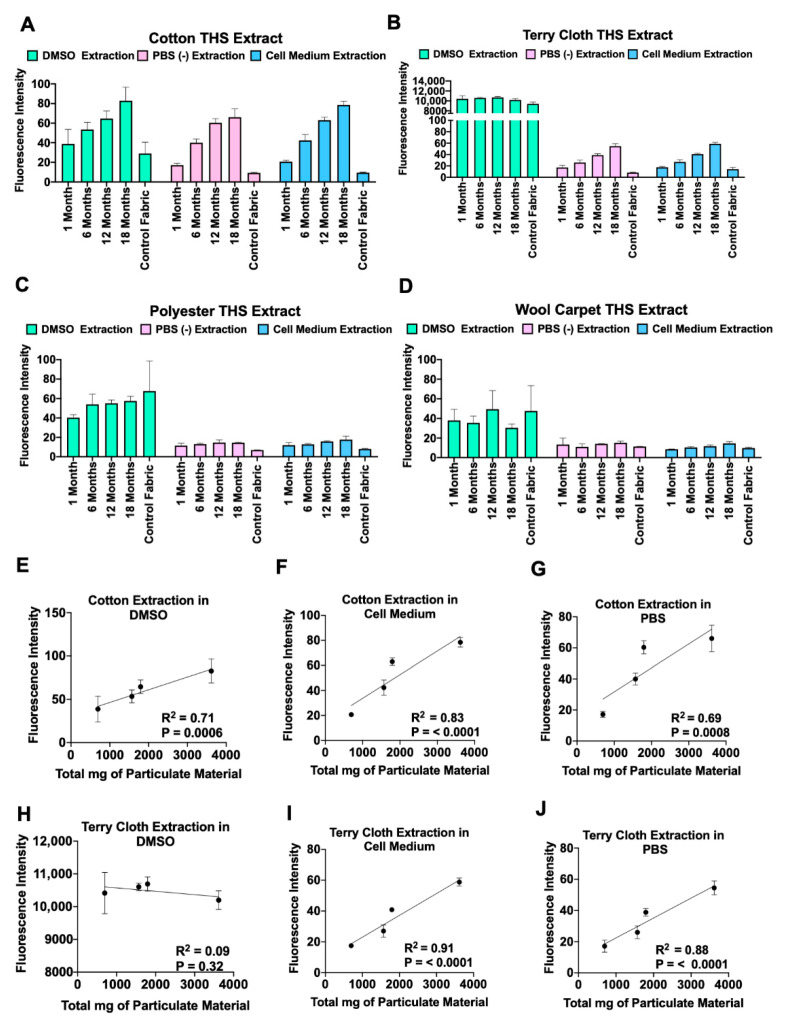
Fluorescence measurements of THS extracts showing that the extracts were autofluorescent. (**A**–**D**) Excitation wavelength 360/40 with emission at 460/40 detected THS residue in fabrics extracted in DMSO, PBS, and cell medium. (**E**–**J**) Fluorescence intensity was proportional to the amount of THS exposure received by the fabrics extracted in PBS and cell medium but not extracts in DMSO.

**Figure 3:**
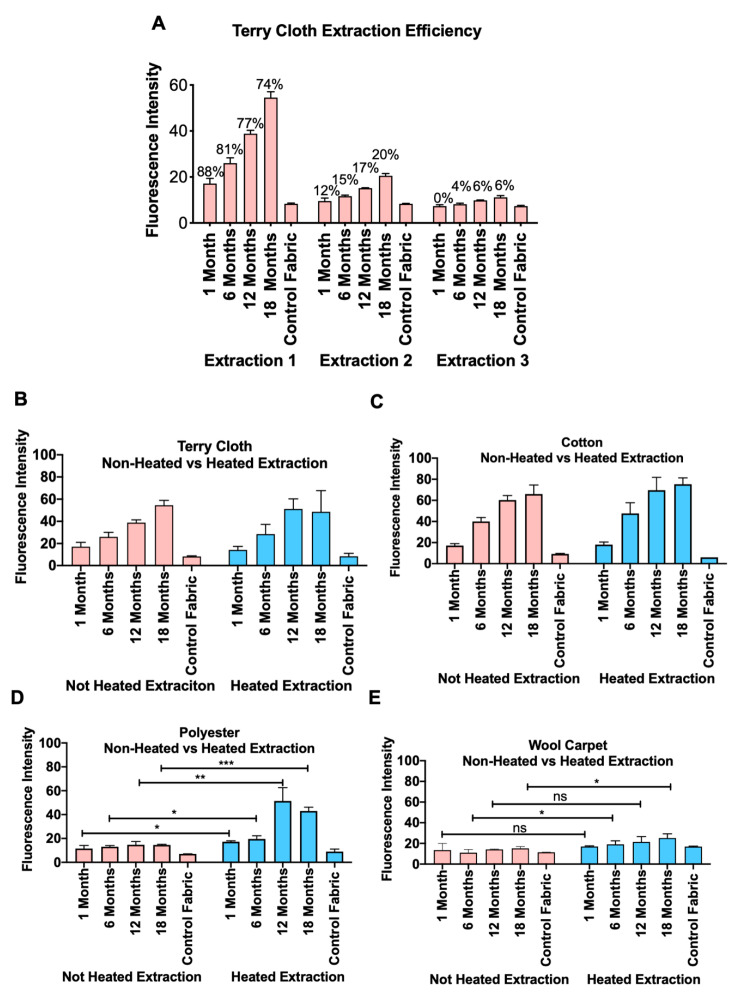
Effect of multiple extractions and heating on extraction efficiency as measured using autofluorescence. (**A**) Most of the residue was removed in the first of three extractions. (**B**–**E**) Extraction was improved in some fabrics by heating for 1 h at 60 °C. (* *p* < 0.05, ** *p* < 0.01, *** *p* < 0.001).

We measured autofluorescence of THS extracts as a quick and efficient method to evaluate THS accumulation in exposed fabrics using various extraction media (Figure 2A–D). While extracts from control fabrics had low levels of autofluorescence, extracts from THS-exposed cotton showed progressively more fluorescence with longer exposures in all extraction media (Figure 2A). Terry cloth produced similar results when extracted in PBS or culture medium; however, DMSO extracts from all groups contained highly autofluorescent material (Figure 2B). THS extracts from polyester and wool carpet showed little fluorescence above the control, and fluorescence did not increase with longer THS exposures (Figure 2C,D). Regression analysis showed a significant correlation between length of exposure and autofluorescence for extracts of cotton fabric in each medium and for terry cloth in PBS and cell culture medium, but not in DMSO (Figure 2). Autofluorescence also proved to be an excellent method to test extraction efficiency (Figure 3A). Using this method, we showed that most (74–89%) THS residue is extracted from terry cloth in the first of three extractions in PBS (Figure 3A).

### 3.3. Heating Increased the Release of THS from Polyester and Wool Carpet

To facilitate the release of THS from polyester and wool carpet, fabrics in PBS were heated to 60 °C for 1 h. Extracts were analyzed using autofluorescence. Heating did not alter the fluorescence intensity between heated and nonheated extracts for both cotton and terry cloth. In contrast, heating facilitated the release of THS from polyester and wool carpet (Figure 3B–E). THS release from polyester was significantly higher in extracts at all time points, while for wool carpet it was only significantly higher for the 6- and 18-month fabric extracts (Figure 3D,E).

### 3.4. Analysis of Chemicals Released into PBS from THS-Exposed Fabrics as Measured Using HPLC

**Figure 4:**
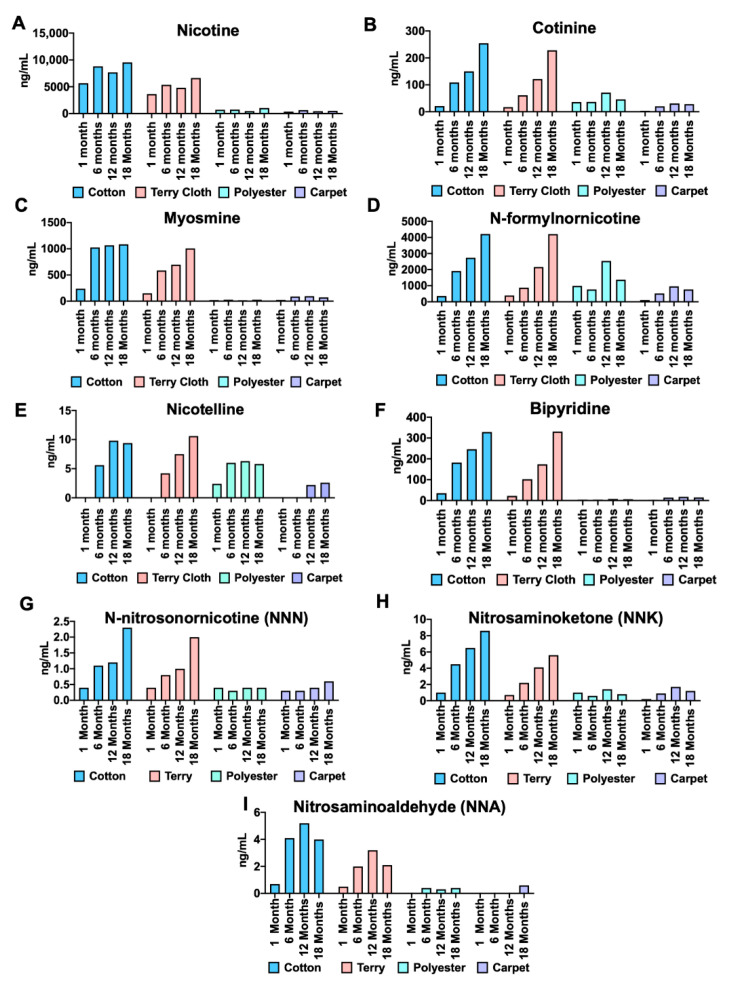
Concentrations of various chemicals in THS extracts of cotton, terry cloth, polyester, and wool carpet measured using HPLC. (**A**–**I**) Nicotine, nicotine alkaloids, and TSNA concentrations were higher in THS extracts from cotton and terry cloth compared to polyester and wool carpet.

Concentrations of nicotine, tobacco alkaloids, and TSNAs were higher in extracts from cotton and terry cloth than those from polyester and wool carpet (Figure 4). Unlike our autofluorescence analysis, nicotine concentrations did not show a progressive increase with exposure for any of the fabrics (Figure 4A). However, concentrations of cotinine, N-formylnornicotine, bipyridine, and NNK did increase progressively with longer exposures for both terry cloth and cotton, but not for polyester or wool carpet (Figure 4). Wool carpet THS extracts from 6-, 12-, and 18-month fabrics had higher concentrations of myosmine, nicotelline, NNA, and NNN than the 1-month extracts, although the relationships were not always linear (Figure 4).

### 3.5. Factors Affecting the Efficiency of Nicotine Extraction from Fabrics

**Figure 5:**
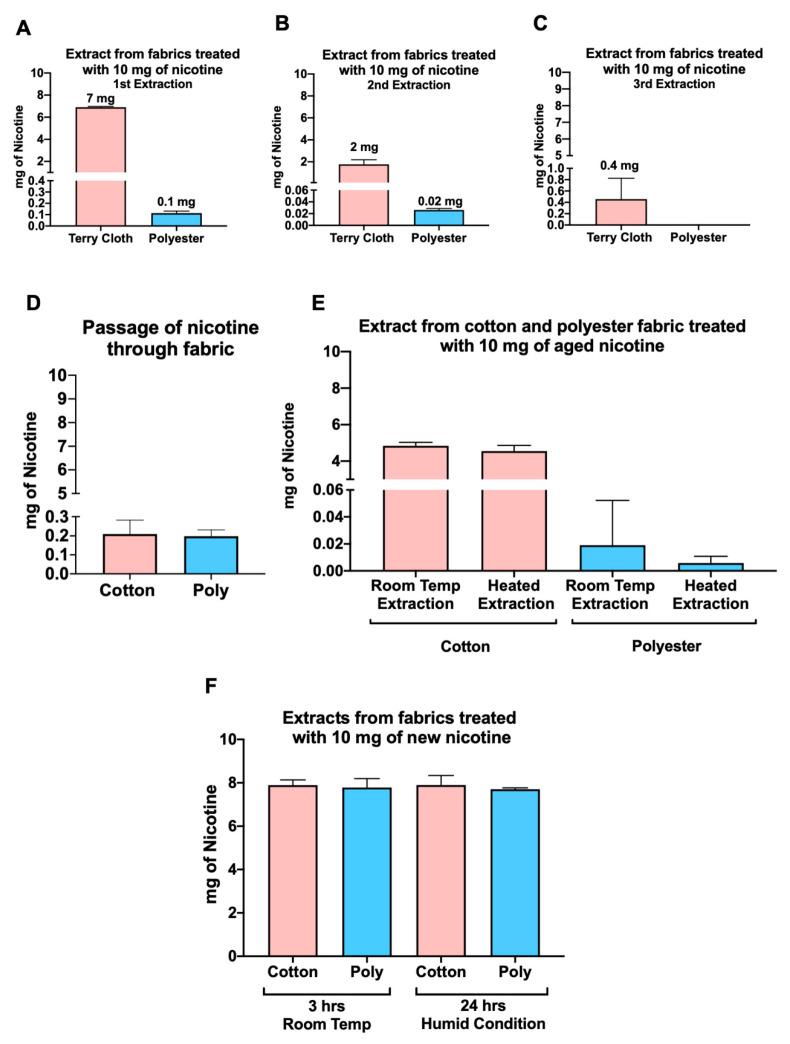
Nicotine extraction was more efficient from polyester when fabrics were exposed in humid conditions. Nicotine was measured using HPLC. Ten milligrams of nicotine was placed on cotton or polyester, and nicotine was extracted in water 24 h later. (**A**–**C**) Most of the nicotine (7 of 10 mg) was recovered in the first extract of terry cloth, while only 0.11 mg was recovered from polyester. (**D**) Nicotine did not filter through either of the fabrics. (**E**) Heating did not improve the release of nicotine from cotton or polyester. (**F**) About 80% of the nicotine was recovered from both cotton and polyester when exposure was done for 3 h at room temperature or for 24 h in humid conditions.

We treated terry cloth and polyester fabric with 10 mg of pure nicotine for 24 h before extracting the nicotine in HPLC unbuffered mobile phase. While extraction in mobile phase rather than PBS may affect the results, we did find that similar to our THS-exposed fabrics, higher concentrations of nicotine were recovered from cotton (5 mg) and terrycloth (7 mg) than from polyester (0.11 mg) (Figure 5A). Most nicotine was recovered in the first extraction, while very little nicotine was recovered in any of the polyester extractions (Figure 5B,C).

Loss of nicotine to the surface below the test fabric was investigated by placing a second piece of cotton fabric beneath the test fabric and measuring nicotine in it. Extracts from the cotton fabric beneath the test fabric had low nicotine concentrations (~0.2 mg total of nicotine), showing that minimal amounts of nicotine leaked through the test fabric (Figure 5D). Nicotine recovery did not differ in heated and nonheated cotton and polyester extracts (Figure 5E).

To test the effect of incubation time on nicotine recovery, we tested a 3-h nicotine exposure. In this assay, nicotine droplets soaked into the fabric, but the fabric did not fully dry out prior to extraction. When the incubation was only 3 h, 80% of the nicotine was successfully recovered from both cotton and polyester, indicating that the time on fabrics and dryness of the fabrics influence extractability of nicotine (Figure 5F). In a third experiment, droplets containing nicotine were added to fabrics that were maintained in a humid environment for 24 h before extraction. An average of 8 mg of nicotine was recovered from both the cotton and polyester that were incubated in high humidity (Figure 5F), indicating that extraction efficiency of nicotine is also dependent on ambient humidity.

## 4. Discussion

Proper assessment and remediation of THS are necessary to reduce exposure for people who cannot move out of THS-contaminated housing. Here we describe a rapid autofluorescence method that can be used to evaluate changes in the THS content of common household fabrics. We have taken advantage of the autofluorescence associated with tobacco tar and total particulate matter [27,28] to develop a rapid assay for quantitatively evaluating THS contamination. Many research facilities have fluorescent spectrometers, and the sample processing, instrument reading, and data analysis time needed for this method is far shorter than that for assays that quantify specific chemicals. We demonstrated an increase in autofluorescence intensity with increased accumulation of THS in fabrics (Figure 2). A linear relationship was observed between the length of time that cotton and terry cloth were exposed to THS and fluorescence intensity in culture medium and PBS extracts. However, extraction of terry cloth with DMSO produced nonlinear results, perhaps because DMSO removed chemical surface treatments, such as fire retardant, that interfered with THS evaluation. It is also possible that the loose weave of the terry cloth tended to dissociate more readily in DMSO than the upholstery cotton. In PBS extracts, results were reproducible, with little variation between extractions of the same fabric, as shown by the small standard deviations (Figure 3A). This assay proved to be a quick, reproducible, and inexpensive method to evaluate THS chemicals in aqueous extracts and a valuable tool to compare or normalize THS extracts between different experiments, batches, or labs. Being able to rapidly compare THS levels in different fabrics or in different labs is currently a hurdle, which may be overcome by taking advantage of this autofluorescence intensity assay.

Autofluorescence was also used to gain insight into THS absorption by cotton, terry cloth, polyester, and wool carpet. Most THS residue was removed from terry cloth after one extraction (Figure 3). Only cotton and terry cloth extracts had a progressive increase in fluorescence intensity with increased length of exposure; however, a significant increase in THS recovery from polyester and wool was obtained by heating during extraction. Previous studies have concluded that polyester fabric does not absorb THS [29,30,31,32]. However, these studies tested THS extraction using aqueous media without heat or by analyzing off-gas chemicals from smoke-exposed fabrics [29,32,33]. Without heat, aqueous extraction and chemical off-gassing might not give complete information on polyester absorption/desorption dynamics. Other studies have shown that polyester fibers gain less weight than cotton when exposed to smoke for 15 min [30,31], which is consistent with our data showing that even after heated extractions, cotton extracts had higher levels of fluorescence than polyester (Figure 3).

Our chemical analysis of THS extracts showed that absorption and extraction are chemical- and fabric-dependent. In agreement with our fluorescence analysis and prior study on THS [6], cotton and terry cloth released higher concentrations of nicotine than polyester and wool carpet. However, nicotine did not increase linearly over time. The lower-than-expected concentrations of these chemicals in the 18-month samples may be due to several factors. First, nicotine can react with atmospheric pollutants, such as ozone and nitrous acid [4,34], which would lower its concentration in the fabric. Second, nicotine can desorb from cotton, as shown by off-gassing analysis [34]. Even though some THS nicotine is likely desorbed and may be chemically converted during aging, microgram concentrations were retained over 18 months. Similar retention of nicotine has been reported in field sites where nicotine was found in smokers’ homes that had been vacant for over 2 months [19].

Like nicotine, myosmine and nicotelline did not increase linearly in cotton extracts but did in terry cloth, suggesting that desorption/conversion of chemicals could be fabric-dependent. Although we did not compare more than two types of cotton, characteristics of the cotton and terry cloth fabric, such as stain release and antiwrinkle treatments, may have contributed to the lack of linear accumulation of myosmine and nicotelline (Figure 4). These treatments affect the off-gassing of volatile organic chemicals in fabrics exposed to cigarette smoke for 15 min [33].

Unlike most chemicals, nicotelline and N-formylnornicotine were extracted from polyester in concentrations similar to those seen in cotton and terry cloth. For nicotelline, polyester was the only fabric extract with detectable concentrations in the 1-month sample. These findings show that (1) some THS alkaloids, such as nicotine and myosmine, turn over during aging of THS either by desorption or chemical conversion; (2) the turnover of myosmine varied with the type of fabric (cotton vs. terry cloth); and (3) some chemicals are readily released from polyester but not from other fabrics (e.g., nicotelline and N-formylnornicotine).

The nitrosamines NNN and NNK, but not NNA, showed linear accumulation over 18 months. While NNN and NNK can remain stable over time, up to 50% of NNA can be lost within the first 2 h after smoking [4]. Our fabrics were exposed for months, allowing off-gassing or chemical reactions of NNA from THS over time. For polyester and carpet, there was little accumulation over time in the extracts, perhaps because TSNAs cannot be extracted from these fabrics or because they are desorbed more readily (Figure 4G–I). This analysis suggests THS chemicals can be absorbed and desorbed at different rates and fabrics play a crucial role in how THS is desorbed.

Our data show that THS can accumulate in household fabrics over time and support the idea that remediation of the affected surfaces is important to prevent adverse health effects. The margin of exposure (MOE) can be used to predict the possibility of cancer developing with prolonged exposure of a toddler to NNK. When a chemical has a MOE <10,000, the risk of developing cancer increases [35,36]. Based on 1 h of mouthing 5 g of cotton fabric exposed to approximately 362 cigarettes for 18 months, a toddler weighing 2.3 kg could be exposed to 0.00016 mg/kg body weight/day of NNK. Using the benchmark dose lower limit (BMDL) of 0.0052 mg/kg body weight/day for NNK [37], MOE can be calculated as MOE = BMDL/estimated exposure dose [38]. The MOE for a toddler exposed to 0.00016 mg/kg body weight/day of NNK is 32. This value, which is well below the threshold of 10,000, indicates that chronic exposure to 0.00016 mg of NNA/kg body weight/day carries with it a risk of developing cancer in the future.

Our data also underrepresent a real environmental THS exposure. The average smoker consumes 14 cigarettes a day [39] or 7560 cigarettes in 18 months versus the ~362 cigarettes used in our 18-month exposed fabrics. In 0.1 g of cotton fabric exposed for 18 months, we recovered 8 ng of NNK. If the same fabric is exposed to 7560 cigarettes, THS could accumulate ~160 ng of NNK per 0.1 g of fabric. Accumulation of THS can occur over many years, which would further increase these numbers. TSNA levels found in dust collected in smokers’ houses have exceeded the lifetime cancer risk recommended by the WHO [40]. Our data support the idea that to protect nonsmokers from TSNAs and other toxicants, remediation methods need to be implemented that are tailored to each fabric.

To further understand differences in the binding affinity of chemicals to cotton, terry cloth, and polyester, all fabrics were exposed to known amounts of nicotine (10 mg/mL) prior to extraction. Nicotine recovery from polyester after a 24-h room temperature incubation was minimal compared to cotton and terry cloth (Figure 5A,B). Although nicotine was completely absorbed by polyester, aqueous extraction did not efficiently remove it. When polyester was treated with nicotine in a container with high humidity for 24 h, 89% of nicotine was recovered (Figure 5F). Equivalent nicotine concentrations were also recovered from cotton and polyester after treating fabrics with nicotine for 3 h (Figure 5F). Clearly, nicotine was absorbed by polyester, but once the fabric was well dried, nicotine became tightly bound and extraction was difficult. This phenomenon could also be true for other THS chemicals, which can be absorbed by polyester but bind tightly and cannot be easily desorbed under dry conditions. THS may be more extractable from nonpolar fabrics like polyester in geographic regions with high humidity, such as Florida.

## 5. Conclusions

We have demonstrated that (1) autofluorescence is an inexpensive, one-step method to quantify THS and (2) absorption and extraction of THS chemicals depend on the chemical of interest, the fabric that it has sorbed to, the temperature of extraction, and the ambient humidity during sorption. THS absorption to fabrics increased with increasing exposure. THS and its associated chemicals were readily extracted from cotton and terry cloth into aqueous media. For polyester, extraction of most chemicals was not efficient unless fabrics were heated or maintained in a humid environment. In contrast with other chemicals, nicotelline was recovered in aqueous extracts of polyester using standard extraction protocols. Our simple, rapid autofluorescence method can be used to compare and normalize THS concentrations in different batches, laboratories, and field sites. In experimental work and remediation, the extraction of THS chemicals is dependent on the chemicals per se, fabric type, heat, and humidity. Understanding the dynamics of THS in fabrics can help set up appropriate remediation policies to protect humans from exposure.

## Figures and Tables

**Table 1:** Smoke exposure for all fabrics.

	Months			
Smoke exposure	1	6	12	18
Total mg of particulate material at each time interval	696	873	226	1833
Total mg of particulate material accumulated over time	696	1569	1795	3917
Total hours of smoke at each time interval	45.5	74.1	30.3	140.3
Total hours of smoke accumulated over time	45.5	119.4	149.7	289.9

## Data Availability

The data that support the findings of this study are available from the corresponding author, upon reasonable request.

## Supplementary Materials

The following are available online at https://www.mdpi.com/article/10.3390/ijerph18073592/s1, Table S1. Cotton, terry, polyester, and wool carpet daily smoke exposure for 1-month exposed fabrics, Table S2. Cotton, terry, polyester, and wool carpet daily smoke exposure for 6-month exposed fabrics, Table S3. Cotton, terry, polyester, and wool carpet daily smoke exposure for 12-month exposed fabrics, Table S4. Cotton, terry, polyester, and wool carpet daily smoke exposure for 18-month exposed fabrics.
