# Supplementary material for: Adhesion and Removal of Thirdhand Smoke from Indoor Fabrics: A Method for Rapid Assessment and Identification of Chemical Repositories
Source: Int J Environ Res Public Health. 2021 Mar 30;18(7):3592. doi: 10.3390/ijerph18073592 (PMC8037229; doi:10.3390/ijerph18073592)
Supplement: Supplementary file 1 [file ijerph-18-03592-s001.pdf]

Supplementary Materials

Table S1. Cotton, terry, polyester and wool carpet daily smoke exposure for 1-month exposed fabrics

Date exposed	Hours of smoke	Total mg particles
9/3/15	3.35	17.58
9/4/15	3.98	49.41
9/10/15	3.82	30.95
9/11/15	3.15	66.10
9/17/15	1.87	13.48
9/18/15	4.33	70.60
9/28/15	4.50	49.61
9/29/15	5.42	84.87
10/1/15	3.13	36.93
10/2/15	5.80	68.44
10/6/15	5.95	207.69

Note: All fabrics were placed inside the smoking chamber on 8/31/15 and removed on 10/8/15

Table S2. Cotton, terry, polyester and wool carpet daily smoke exposure for 6-month exposed fabrics

Date exposed	Hours of smoke	Total mg particles
9/3/15	3.35	17.58
9/4/15	3.98	49.41
9/11/15	3.15	66.10
9/17/15	1.87	13.48
9/18/15	4.33	70.60
9/28/15	4.50	49.61
9/29/15	5.42	84.86
10/1/15	3.13	36.93
10/2/15	5.80	68.44
10/6/15	5.95	207.69
10/12/15	4.55	39.57
10/13/15	5.70	123.83
10/15/15	4.13	40.10
10/19/15	5.45	60.12
10/20/15	3.85	60.60

10/22/15	6.03	62.29
10/23/15	5.88	71.95
10/26/15	6.13	72.63
10/27/15	5.57	67.89
10/29/15	5.92	54.49
11/6/15	3.12	33.94
11/20/15	3.13	31.97
11/24/15	3.08	30.79
1/8/16	4.22	52.50
1/29/16	3.18	25.55
2/5/16	4.10	45.62

Note: All fabrics were placed inside the smoking chamber on 8/31/15 and removed on 3/8/16

Table S3. Cotton, terry, polyester and wool carpet daily smoke exposure for 12-month exposed fabrics

Date exposed	Hours of smoke	Total mg particles
9/3/15	3.35	17.58
9/4/15	3.98	49.41
9/10/15	3.82	30.95
9/11/15	3.15	66.10
9/17/15	1.87	13.48
9/18/15	4.33	70.60
9/28/15	4.50	49.61
9/29/15	5.42	84.87
10/1/15	3.13	36.93
10/2/15	5.80	68.44
10/6/15	5.95	207.69
10/12/15	4.55	39.57
10/13/15	5.70	123.83
10/15/15	4.13	40.06
10/19/15	5.45	60.12
10/20/15	3.85	60.60
10/22/15	6.03	62.29
10/23/15	5.88	71.95
10/26/15	6.13	72.63

10/27/15	5.57	67.89
10/29/15	5.92	54.49
11/6/15	3.12	33.94
11/20/15	3.13	31.97
11/24/15	3.08	30.79
1/8/2016I7	4.22	52.50
1/29/16	3.18	25.55
2/5/16	4.10	45.62
3/10/16	4.57	46.34
3/31/16	4.28	45.84
4/7/16	4.75	27.76
4/21/16	3.85	65.13
4/26/16	2.23	16.56
4/29/16	1.77	10.33
6/3/16	2.55	2.42
7/8/16	3.15	4.41
8/12/16	3.15	6.43

Note: All fabrics were placed inside the smoking chamber on 8/31/15 and removed on 9/7/16

Table S4. Cotton, terry, polyester and wool carpet daily smoke exposure for 18-month exposed fabrics

Date exposed	Hours of smoke	Total mg particles
9/3/15	3.35	17.58
9/4/15	3.98	49.41
9/10/15	3.82	30.95
9/11/15	3.15	66.10
9/17/15	1.87	13.48
9/18/15	4.33	70.60
9/28/15	4.50	49.61
9/29/15	5.42	84.86
10/1/15	3.13	36.93
10/2/15	5.80	68.44
10/6/15	5.95	207.69
10/12/15	4.55	39.57
10/13/15	5.70	123.83
10/15/15	4.13	40.10
10/19/15	5.45	60.12

10/20/15	3.85	60.60
10/22/15	6.03	62.30
10/23/15	5.88	71.95
10/26/15	6.13	72.63
10/27/15	5.57	67.89
10/29/15	5.92	54.49
11/6/15	3.12	33.94
11/20/15	3.13	31.98
11/24/15	3.08	30.79
1/8/2016I7	4.22	52.50
1/29/16	3.18	25.55
2/5/16	4.10	45.62
3/10/16	4.57	46.34
3/31/16	4.28	45.84
4/7/16	4.75	27.76
4/21/16	3.85	65.13
4/26/16	2.23	16.56
4/29/16	1.77	10.33
6/3/16	2.55	2.42
7/8/16	3.15	4.41
8/12/16	3.15	6.430
10/20/16	5.10	24.68
10/27/16	6.07	20.54
11/3/16	5.87	110.75
11/10/16	5.50	86.79
11/17/16	5.02	79.69
11/18/16	4.12	49.12
11/23/16	5.90	79.39
11/29/16	5.78	67.77
12/2/16	6.07	56.36
12/8/16	5.92	51.54
12/16/16	5.67	71.62
12/22/16	6.13	88.80
1/13/17	5.00	84.85
1/20/17	5.17	139.98
1/26/17	4.98	115.73
1/27/17	5.07	96.45
2/2/17	5.05	60.147

2/3/17	4.42	50.998
2/10/17	5.00	49.846
2/15/17	5.12	79.520
2/17/17	5.07	74.045
2/22/17	5.73	103.440
2/24/17	5.58	114.076
3/1/17	5.68	128.603
3/2/17	5.98	121.058
3/3/17	5.28	116.510

Note: All fabrics were placed inside the smoking chamber on 8/31/15 and removed on 3/7/17
